# Learning the effects of psychotropic drugs during pregnancy using real-world safety data: a paradigm shift toward modern pharmacovigilance

**DOI:** 10.1007/s11096-018-0672-2

**Published:** 2018-06-15

**Authors:** Angela Lupattelli, Olav Spigset, Hedvig Nordeng

**Affiliations:** 1 PharmacoEpidemiology and Drug Safety Research Group, School of Pharmacy, and PharmaTox Strategic Research Initiative, Faculty of Mathematics and Natural Sciences, University of Oslo, Oslo, Norway; 2 Department of Clinical Pharmacology, St Olav’s University Hospital, Trondheim, Norway; 3 Department of Clinical and Molecular Medicine, Norwegian University of Science and Technology, Trondheim, Norway; 4 Department of Child Health and Development, Norwegian Institute of Public Health, Oslo, Norway

**Keywords:** Pharmacovigilance, Pregnancy, Psychotropic drugs, Real-world data, Safety

## Abstract

The growing evidence on psychotropic drug safety in pregnancy has been possible thanks to the increasing availability of real-world data, i.e. data not collected in conventional randomised controlled trials. Use of these data is a key to establish psychotropic drug effects on foetal, child, and maternal health. Despite the inherent limitations and pitfalls of observational data, these can still be informative after a critical appraisal of the collective body of evidence has been done. By valuing real-world safety data, and making these a larger part of the regulatory decision-making process, we move toward a modern pregnancy pharmacovigilance. The recent uptake of real-world safety data by health authorities has set the basis for an important paradigm shift, which is integrating such data into drug labelling. The recent safety assessment of sodium valproate in pregnant and childbearing women is probably one of the first examples of modern pregnancy pharmacovigilance.

## Introduction

Perinatal psychiatric disorders occur in one out of five women, and among these, a substantial number may require treatment with psychotropic drugs, even during pregnancy [1]. Gestational use of these drugs has been on the rise in the last decades. For instance, the prevalence of antidepressant use increased from 1% in the nineties, to a current 3% in Europe and 8% in the USA [2, 3]. Hence, addressing the safety profile of psychotropics in pregnancy has become an important public health concern.

Pharmacotherapy with psychotropic drugs during pregnancy involves weighing the possible risk of foetal exposure to the drug against the potential adverse effects of sub-optimally treated maternal psychiatric illness to both the mother and child. To guide such decisions, it is critical to provide sound data about psychotropic drug safety in pregnancy and to appraise the collective body of evidence. However, benefits cannot be weighed against risks before reliable information on safety for both immediate perinatal (e.g., congenital anomalies) and long-term (e.g. neurodevelopmental) outcomes in the offspring are available.

In this commentary, we discuss the value of real-world drug safety data in pregnancy, i.e. data not collected in conventional randomised controlled trials [4] but rather via observational, pharmacoepidemiological investigations. We also address the methodological advances and challenges linked to use of these data, with focus on psychotropic drugs. Finally, we consider the translation of this safety information into clinical guidance, as a shift toward a “modern” pregnancy pharmacovigilance.

## The importance of pharmacoepidemiological pregnancy studies

Until now results from reproductive in vivo and in vitro toxicity testing, coupled to human case-reports and series, and observational post-marketing studies, have aided our understanding of the potential risks posed by psychotropic drug exposure in pregnancy. Yet, we are now witnessing a major transition in the way pharmacoepidemiological pregnancy studies are valued as a methodological key to provide real-world evidence on drug safety in pregnancy. Not least, the recent utilization of data from observational studies by the health authorities has also set the basis for an important paradigm shift, which is providing meaningful clinical information about human drug exposure during pregnancy to women and their doctors [5].

## Benefits and challenges of real-world data

In the last two decades there has been an important, rapid escalation of published data on human foetal safety following in utero exposure to psychotropic drugs. Most studies have explored risks of immediate perinatal outcomes such as congenital anomalies, foetal death, and poor neonatal adaptation [6], however the research focus has recently shifted toward various longer-term developmental outcomes in the offspring, such as cognition, neuromotor and behavioural effects, attention-deficit hyperactive disorder, and autism spectrum disorder.

The accumulating, growing evidence on psychotropic drug safety in pregnancy has been possible thanks to the increasing availability of real-world data. These include, among others, pregnancy cohort studies and registries, research consortia, health registries, administrative databases and direct-to-patient research initiatives [7]. Use of real-world data is a key to establish psychotropic drug effects on foetal, child, and maternal health. At the same time, the observational nature of real-world data entails inherent limitations and pitfalls, i.e. confounding, bias, and chance, that need to be dealt with.

Assignment of a psychotropic drug in pregnancy is neither random nor blinded, and so women taking these drugs differ from non-users in a variety of ways which are often difficult to control for and/or hard to measure (e.g., psychiatric disease severity, illicit substance use), or that remain completely unmeasured (e.g., genetic susceptibility, familial environment). However, we are at a crucial moment where ‘measured’ confounding can be limited in pregnancy studies by the application of novel statistical methods (e.g., propensity scores) [8]. Use of augmented real-world data can also help us to limit the bias due to exposure or outcome misclassification, e.g. by ascertaining psychotropic drug exposure in pregnancy via multiple sources. Direct-to-patient studies can provide valuable, granular data on women’s mental health, behaviours and drug exposures at multiple time points during gestation, which are often lacking in registry-based and administrative data studies [7]. Although real-world data are observational by definition, the design of a hypothetical randomized clinical trial can still be conceptualized [9]. This strategy can allow fairer comparisons between those women exposed to psychotropic drugs in pregnancy and those who are not, and thus reduce, at least to some extent, differences in severity of the underlying psychiatric disease. All these strategies, coupled to methods to address the impact of ‘unmeasured’ confounding (e.g., sibling-designs) [8] can enable us to get closer to the ‘true’ psychotropic drug effects on maternal and child health. To reach this goal, though, we are often in need of large multinational registry data, which, beyond offering the statistical power to apply sibling-designs, provide the additional benefit to explore the safety of individual psychotropics during pregnancy [10].

Generally, association does not imply causation, but real-world data on psychotropic drug safety in pregnancy can be informative after a critical appraisal of the available evidence has been done. Important factors include, among others: the strength and direction of the association exposure-outcome and its replication across studies; the specificity of the association; the temporal and dose–response relationships; and not least biological plausibility. Appraising the prevalence of both the psychotropic drug and the outcomes of interest, remains crucial from a public health perspective. For instance, even the large relative increase in the risk of persistent pulmonary hypertension of the newborn associated with prenatal antidepressant exposure, would translate, clinically, into a small absolute risk [10].

## Modern pregnancy pharmacovigilance?

Several activities are parts of the puzzle for a modern pregnancy pharmacovigilance, i.e. a pharmacovigilance system that makes real-world data a larger part of the regulatory decision-making process. The EUROmediCAT consortium in Europe, for instance, can provide important insights into potential safety signals in pregnancy in the early post-marketing stage [11]. The initiatives by the European Teratology Information Services [12] have, among others, the ability to collect observational pregnancy data on rare drug exposures with insufficiently documented safety information, e.g., antipsychotics. High standard, high quality, and high transparency post-authorization, pharmacoepidemiological pregnancy studies, are additional core factors to strengthen, as recently advocated in Europe in relation to the detrimental developmental effects of antiepileptic drugs in pregnancy on the offspring [5, 13].

Indeed, the reproductive safety of sodium valproate, an antiepileptic drug also used for treatment of bipolar disorders, was recently assessed by the Pharmacovigilance Risk Assessment Committee within the European Medicine Agency (EMA) [14]. Sodium valproate was also just banned by the French National Agency for the Safety of Medicines and Health Products for use by pregnant and childbearing-age women, specifically those with bipolar disorders [15]. This measure was undertaken in light of the now substantial evidence about the detrimental effects of prenatal sodium valproate in pregnancy on child health, in terms of both congenital anomalies and neurodevelopmental delays [16, 17]. Nevertheless, while alternative treatments may be available to women with bipolar disorders, this is often not the case for epilepsy, remarking the importance of the maternal underlying disorder when assessing the benefit-risk ratio of drugs in pregnancy.

Modern pregnancy pharmacovigilance also entails conveying real-world evidence in a regulatory actionable and clinically meaningful way, i.e. integrate these data into drug labelling. The latter point is indeed of importance, and advances have been made in both Europe and the USA in recent years. In the USA, removal of the pregnancy risk category letter system in favor of a narrative structure, which includes real-world safety information about dosing and fetal risks [18], has represented a crucial step forward. In Europe, the *Guideline on risk assessment of medicinal products on human reproduction and lactation: from data to labelling* [19] by the EMA has also supported the need to update the recommendations for use during pregnancy and lactation in light of the increasing human experience in exposed pregnancies.

However, the potential risks posed by a sub-optimally medicated maternal illness during pregnancy on child and maternal health are insufficiently conveyed, as this information is not part of the drug labelling. Therefore, the question remains as to how pregnant women with psychiatric disorders, for instance, can be empowered to take informed clinical decisions about the benefits and potential risks of psychotropic drug use during pregnancy.

Despite all the advances, modern pregnancy pharmacovigilance activities should also endorse involvement of pregnant and childbearing-aged women in research, as well as in public hearings, as recently done by the EMA in relation to sodium valproate use in pregnancy [20]. Efforts should be made to enhance use of patient-generated health data in pharmacoepidemiological pregnancy studies and direct-to-patient research approaches, and to re-think the way psychotropic drug exposure in pregnancy has so far been defined and studied. Indeed, it is crucial to understand how different patterns of psychotropic drug exposure throughout pregnancy, based on intensity and duration of drug use, may negatively impact maternal and child health. Likewise, estimating direct drugs effects, i.e. effects that go beyond those posed by intermediate pre-or postnatal factors, and quantifying the potential detrimental effects posed by the underlying psychiatric disorder if not treated adequately, has become imperative for an ultimate rational use of drugs in pregnancy.

Real-world safety data on psychotropics in pregnancy and their incorporation into labelling constitute an important first shift toward a modern pharmacovigilance system for maternal-child health. This is essential for clinical guidance on treatment options and evidence-based counselling to perinatal women with severe psychiatric disorders.
