# Correction to: Learning the effects of psychotropic drugs during pregnancy using real‑world safety data: a paradigm shift toward modern pharmacovigilance

**DOI:** 10.1007/s11096-020-01104-2

**Published:** 2020-07-25

**Authors:** Angela Lupattelli, Olav Spigset, Hedvig Nordeng

**Affiliations:** 1 PharmacoEpidemiology and Drug Safety Research Group, School of Pharmacy, and PharmaTox Strategic Research Initiative, Faculty of Mathematics and Natural Sciences, University of Oslo, Oslo, Norway; 2 Department of Clinical Pharmacology, St Olav’s University Hospital, Trondheim, Norway; 3 Department of Clinical and Molecular Medicine, Norwegian University of Science and Technology, Trondheim, Norway; 4 Department of Child Health and Development, Norwegian Institute of Public Health, Oslo, Norway

## Correction to: International Journal of Clinical Pharmacy (2018) 40:783–786 10.1007/s11096-018-0672-2

The article Learning the effects of psychotropic drugs during pregnancy using real‑world safety data: a paradigm shift toward modern pharmacovigilance, written by Angela Lupattelli · Olav Spigset · Hedvig Nordeng, was originally published Online First without Open Access. After publication in volume 40, issue 4, page 783-786 the author decided to opt for Open Choice and to make the article an Open Access publication. Therefore, the copyright of the article has been changed to © The Author(s) 2018 and the article is forthwith distributed under the terms of the Creative Commons Attribution 4.0 International License (http://creativecommons.org/licenses/by/4.0/), which permits use, duplication, adaptation, distribution and reproduction in any medium or format, as long as you give appropriate credit to the original author(s) and the source, provide a link to the Creative Commons license, and indicate if changes were made.

